# The epidemiology of inflammatory rheumatic diseases in Germany in a European context: a systematic review to support workforce planning

**DOI:** 10.1016/j.ero.2026.100208

**Published:** 2026-07-09

**Authors:** Katinka Albrecht, Carlo A.G. Veltri, Anja Strangfeld, Johanna Callhoff

**Affiliations:** 1 German Rheumatology Research Center, Epidemiology and Health Services Research, Berlin, Germany; 2 Department of Rheumatology and Clinical Immunology, Charité-Universitätsmedizin Berlin, Berlin, Germany

## Abstract

**Objectives:**

The number of adults living with inflammatory rheumatic diseases (iRMDs) in a country is key for calculating the demand for rheumatologists. Our aim was to estimate the incidence and prevalence of iRMD in Germany by compiling current epidemiologic German and other European data.

**Methods:**

A systematic review included original studies on prevalence and incidence of iRMDs in Germany and other European countries, covering study data from 2013 to the present. Considering heterogeneous methodology, we qualitatively estimated the prevalence and incidence for 11 iRMDs and extrapolated the results to the German population.

**Results:**

A total of 20 German and 72 studies from other European countries were considered. All German studies used claims data with moderate to high risk of bias. Studies relying solely on *International Classification of Diseases* codes as case definition reported higher rates than those applying diagnostic criteria or multistage population-based methods. Prevalence ranges (with estimates for Germany in parentheses) per 100,000 are: rheumatoid arthritis 410 to 1380 (1000), polymyalgia rheumatica 100 to 937 (600 aged >50 years), axial spondyloarthritis 75 to 500 (300), psoriatic arthritis 20 to 580 (300), giant cell arteritis 76 to 250 (146 aged >50 years), primary Sjögren's disease 32 to 250 (70), systemic lupus erythematosus 35 to 210 (56), systemic sclerosis 17 to 50 (35), idiopathic inflammatory myopathies 17 to 25 (17), anti-neutrophil cytoplasmic antibody-associated vasculitides 14 to 43 (26), and Behçet’s disease 0.3 to 15 (4.7). Overall, we estimate the prevalence of iRMD in Germany to be 2.6% (1.8 million adults) and an annual incidence of 0.2% (130,000 adults).

**Conclusions:**

This study compiles European epidemiological data on 11 iRMDs to calculate the number of people affected. The methodology could be a blueprint for studies in other regions.

## Graphical abstract

**Figure:**
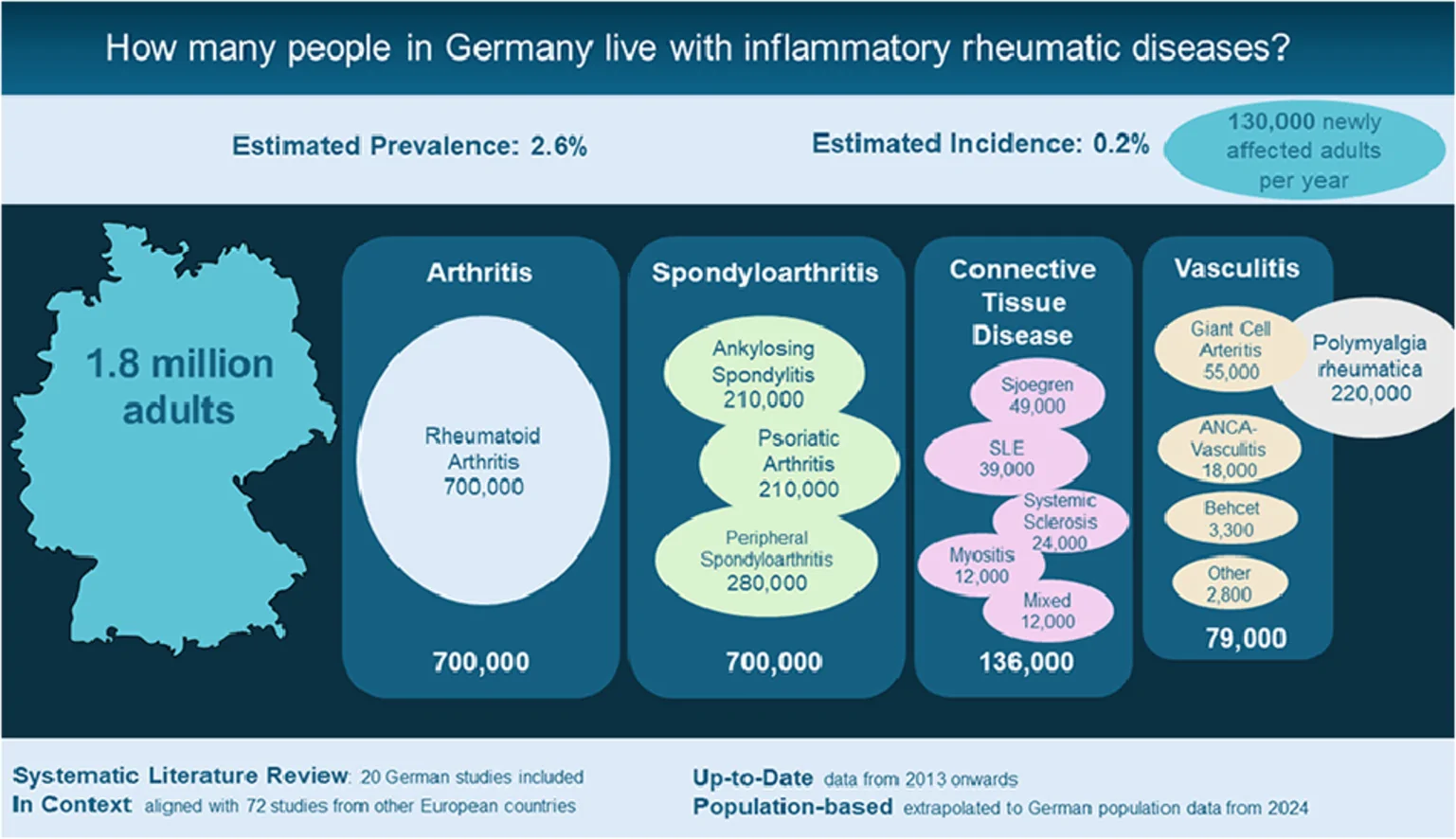

**WHAT IS ALREADY KNOWN ON THIS TOPIC**

For individual inflammatory rheumatic diseases (iRMDs), systematic reviews and meta-analyses report prevalence and incidence rates across countries, but there are no estimates for the total number of people with iRMDs in a single country.

**WHAT THIS STUDY ADDS**

A systematic qualitative assessment of German and other European data provides prevalence and incidence estimates for the most common iRMDs in Germany.

**HOW THIS STUDY MIGHT AFFECT RESEARCH, PRACTICE OR POLICY**

The number of people with prevalent and incident iRMDs in a country are essential prerequisites for rheumatology workforce planning.

Alt-text: Unlabelled box dummy alt text

## INTRODUCTION

Given the global shortage of rheumatologists, the number of people living with inflammatory rheumatic diseases (iRMDs) in a country is crucial for calculating the demand for the rheumatology workforce. Worldwide, we are facing a declining rheumatologist workforce, driven by high expected retirement rates and an increasing proportion of part-time work [[1], [2], [3]]. At the same time, the prevalence of iRMDs is rising [[4], [5], [6]]. This trend can be attributed to several factors, including population aging, an increase in autoimmune diseases (AIDs), improved early diagnoses, and declining mortality. Although systematic reviews on the prevalence and incidence of single iRMDs exist, comprehensive estimates of the total number of people living with iRMDs are lacking. Within the RheumaFacts project, the European Alliance of Associations for Rheumatology (EULAR) is currently working to provide cross-border figures on health care gaps, disease burden, and inequalities among people with iRMDs [7]. So far, there is no systematic compilation of epidemiological European data, and it is difficult to collect such data, as systematic reviews and meta-analyses usually comprise only one iRMD with differing study periods, regions, and methodology, resulting in wide prevalence ranges [[8], [9], [10], [11], [12]]. In 2022, a systematic review on the prevalence of iRMDs in Germany was conducted to estimate the number of people living with iRMDs [4]. Since then, new evidence has emerged, and the population structure has continued to evolve. The aim of the present study was to update these previous estimates and, for the first time, to also assess the incidence of iRMDs based on a systematic review. To place the German data in the European context, a systematic data collection from other European countries served as a basis for comparison.

## METHODS

### Search strategy

A systematic review was performed in PubMed and Web of Science databases from January 2015 to November 2025. Original full-text papers published in German or English language were included, providing data on the prevalence and/or incidence of any iRMD in adults in Germany. Original data from 2013 to the present were eligible. Modeled estimates and survey data with nonrepresentative age groups were excluded. Representative age groups included ages ≥18 years for all iRMDs and ≥50 years for polymyalgia rheumatica (PMR) and giant cell arteritis (GCA).

A predefined search strategy from the former systematic review [4] was adapted including terms related to prevalence and incidence of rheumatoid arthritis (RA), axial spondyloarthritis (axSpA), psoriatic arthritis (PsA), systemic lupus erythematosus (SLE), systemic sclerosis (SSc), idiopathic inflammatory myopathies (IIMs), Sjögren’s disease (SjD), PMR, GCA, anti-neutrophil cytoplasmic antibody (ANCA)-associated vasculitis (AAV), and Behçet disease (BD). The detailed search strategy and process are reported in the Supplementary Material. The literature was independently reviewed by 2 authors (KA, JC). Disagreements were resolved by discussion.

The quality of all included studies was assessed using the checklist by Hoy et al. [13] for reporting prevalence data. For each study, 4 aspects of external validity and 5 aspects of internal validity were evaluated. Each aspect was rated on 2 levels, indicating either a high or low risk of bias. Based on these ratings, an overall judgment of risk of bias was determined and categorized as high, moderate, or low.

### Data extraction

The following data were extracted: first author, publication year, study period, data source, sample size, age inclusion, case definition, *International Classification of Diseases, Tenth Revision, German Modification* (ICD-10-GM) codes, prevalence, incidence per 100,000 (95% confidence interval), and standardization. If several years were reported, the most recent one was selected.

### Comparison with data from other European countries

Original non-German European data from 2013 to the present were searched for the same iRMD (“prevalence or incidence” AND diagnosis [title]). Studies were also extracted from systematic reviews or meta-analyses with data from 2013 to the present. Data sources were assigned to population-based versus health administrative data, including insurance data, electronic medical records, hospital-based records, and disease registries [14].

### Qualitative estimation

The qualitative estimation was conducted by the first and last authors (KA, JC) and reviewed by the head of the epidemiology unit (AS); all are well-versed in epidemiology and experienced in the methodological handling of prevalence data derived from claims data. For each iRMD, we evaluated the German studies in terms of quality and in the context of studies from other European countries. Based on this, we estimated the prevalence and incidence for each iRMD for Germany. The following questions were used for the assessment: (1) Is there evidence from one or more (independent) studies? (2) How does the risk of bias in the available studies affect the results? (3) How well does the evidence align with other European and previous German data? (4) Are there any other reasons to believe that the reported prevalence/incidence are too high or too low? For each iRMD, the assessment process is reported in the results. We prioritized standardized over raw data, nationwide over regional data, comprehensive nationwide data over data from individual health insurance companies, and data with multiple case definitions over the exclusive use of ICD codes. The subjective reliability of the estimates was assessed as low/moderate/good according to the authors’ discretion. Because the studies differ greatly in terms of their methodology, particularly the inclusion criteria and case definition, and do not provide independent samples (several publications are based on subsamples from the same database), double counting occurs, which distorts the prevalence and artificially reduces the variance. We therefore could not perform a random effects meta-analysis. A comparison with data from other European countries was performed as robustness check to support qualitative assessments.

### Calculation of the number of adults

The number of adults with prevalent and incident iRMDs were calculated by extrapolating the results to the German adult population in 2024 [15].

This article was written in accordance with the PRISMA reporting guidelines for systematic reviews [16]. The systematic review is registered in Open Science Framework (OSF), https://doi.org/10.17605/OSF.IO/HVWS9.

### Public/patient involvement

No research partners were involved in this study.

## RESULTS

### Study characteristics

Of 181 articles identified, 14 met the inclusion criteria, and 8 were supplemented by manual search, leading to 22 eligible publications from 20 studies (Fig 1). The extracted data are reported in Table 1 [[17], [18], [19], [20], [21], [22], [23], [24], [25], [26], [27], [28], [29], [30], [31], [32], [33], [34], [35], [36], [37], [38]].

**Figure 1:**
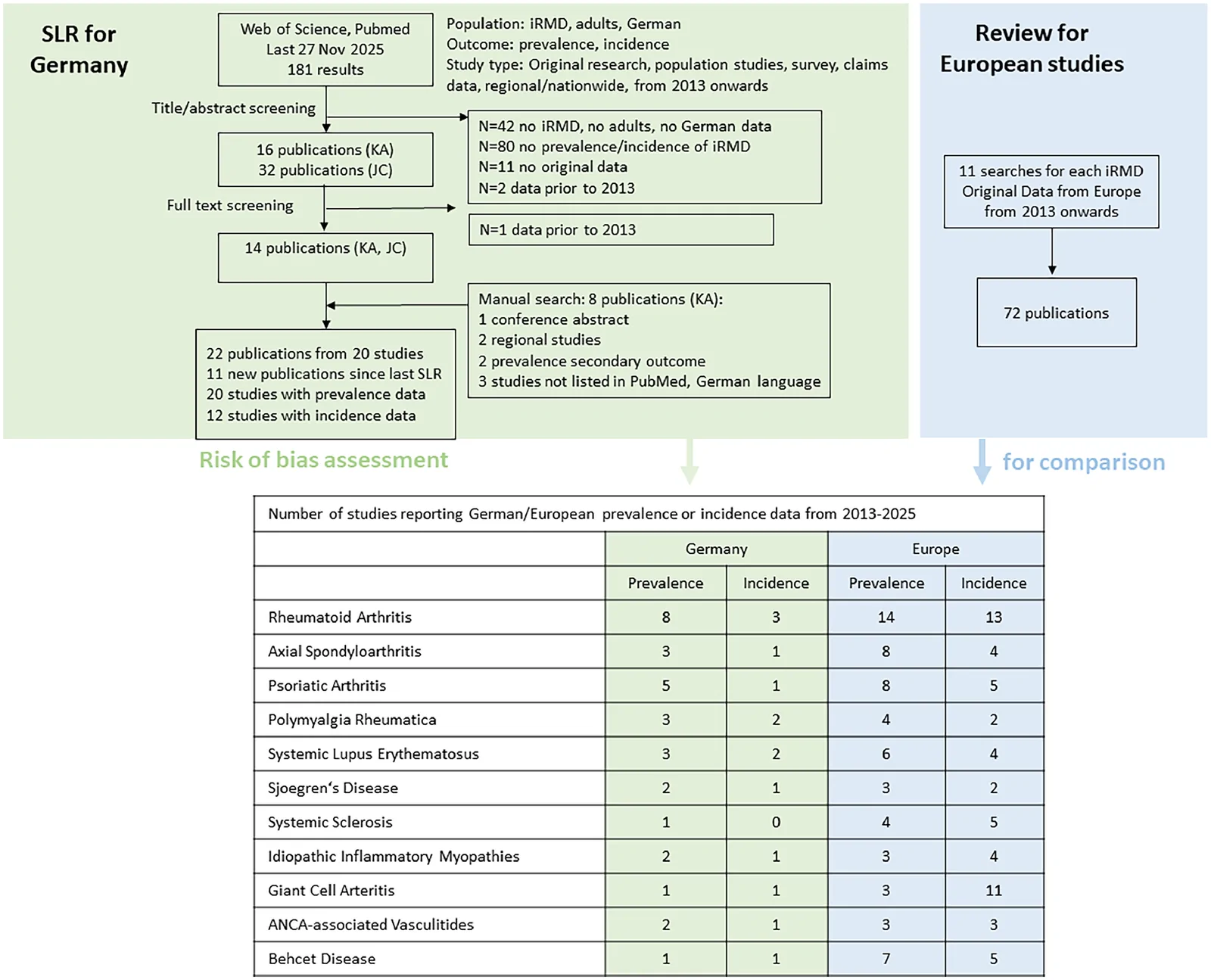
Flow chart. ANCA, anti-neutrophil cytoplasmic antibody; iRMD, inflammatory rheumatic disease.

**Table 1:** Included articles from the systematic review

First author	Publication year	Study period	Data source	Sample size	Age group included	Case definition	ICD-10 codes	iRMD	Prevalence per 100,000	Incidence per 100,000	Standardisation	Estimated number of people affected
Zouboulis [17]	2025	2016-2018	Nationwide SHI (InGef)	4 m	≥18	G, M2Q	M35.2	BD	4.7 [4.0-6.4]	0.6 [0.3-2.1]		
Schmidt [18]	2025	2018-2021	Nationwide SHI (InGef)	4.8 m	≥50	G, M2Q	M35.3, M31.5	PMR	937	111	by age and sex to German population data from 2021	351,340
Schmidt [19]⁎	2026						M31.5, M31.6	GCA	146	23.6		54,934
Alexander [20]	2025	2011-2019	Nationwide SHI (WIG2)	n.r.	any	G, M2Q+lab/med/ specialist/organ	M32.1, M32.8, M32.9	SLE	60	8.86		49,800
Akmatov [21,22]	2024, 2025	2022	Nationwide SHI (ZI)	69-73 m	any	G, M2Q	M05, M06	RA	1357 **(1300)**		by age and sex to German population data from 2022	994,164
							M45	axSpA	290 **(280)**			212,296
							L40.5, M07.0-3	PsA	20 **(20)**			17,999
							M08, M09	JIA	46 **(50)**			34,560
							M35.0	SjD	355 **(330)**			260,161
							M34	SSc	50 **(50)**			35,862
							M32.1/M32.9	SLE	46 **(40)**			37,865
							M33	DM/PM	17 **(17)**			12,782
							M31.5, M31.6, M35.3	PMR/GCA	420 **(400)**			306,960
							M31.3	GPA	20 **(20)**			13,437
WidO [23]	2025	2023	Nationwide SHI (AOK)	27 m	any	G, M2Q	M05, M06	RA	1190			1,008,000
Riemekasten [24], Leipe [25]	2024	2018	Nationwide SHI (InGef)	4 m	any	M2Q	M05, M06	RA	1376 [1365;1387]		No standardisation	
							L40.5, M07	PsA	288 [283;293]			
							M45, M46	axSpA	296 [291;301]			
							M32-35, no M35.3	CTD	785 [464;1098]			
Hagenström [26]	2024	2017-2019	Nationwide SHI (TK)	10.5 m	any	M2Q	L40.5, M07.0-3, M09.0	PsA	350 [350]		by age, sex and SHI region to the population of all SHI-insured persons	230-300,000
Pawlitzki [27]	2022	2005-2019	Nationwide SHI (BARMER)	8.7 m	any	G	M33.0, M33.1, M33.9	DM	11.3	2.5	by age and sex, extrapolated to population data from 2019	8,000
							M33.2	PM	12.5	1.5		8,600
							M60.1	IIM	1.1			820
							M60.8, M60.9, G72.4	Other	39.7			30,500
Colombo [28]	2022	2011-2019	Regional SHI (AOK)	n.r.	≥40	G, M2Q	M35.3, M31.5	PMR	153 (145)	17.7	by age and sex to total SHI population 2019	
Schwarting [29]	2021	2009-2014	Nationwide SHI (BKK)	4.1 m	≥18	G, M2Q+lab/med/specialist	M32.1,8,9	SLE	47.4 (48.5)		adjustment for right-censored data: 55.8	
Kienitz [30]	2021	2013	Nationwide SHI (Vilua)	2.3 m	≥18	G, M2Q	M05, M06	RA	1340		No standardisation	
						M2Q+specialist diagnosis			1070			
						M2Q+med			540			
Hellmich [31]	2021	2013-2016	Nationwide SHI (InGef)	3 m	≥18	G, M2Q	M31.3, M31.7	AAV	25.6 (1.1)		assumed to be representative	
							M31.3	GPA	21.0 (0.7)			
							M31.7	MPA	4.6 (0.4)			
Grellmann [32]	2021	2016	Nationwide SHI (Vilua)	1.9 m	≥18	G	M05.8, M06.0, M06.8	RA	410	0.1-0.2	by age and sex to the total SHI population in the respective year	
							M07.0-3, L40.5	PsA	270-320	0.1		
Albrecht [33]	2020	2018	Nationwide SHI (BARMER)	7.2m	≥18	M2Q +rheumatologist, no AID +rheumatologist + AID	M35.0	SjD (all) pSjD sSjD	750 35 48	100 4.2 4.1	no standardisation	
Strahl [34]	2018	2013	Regional SHI (AOK)	3.4m	≥18	G, M2Q	M05, M06	RA	1050		by age to European standard population of 1976	
						G, M2Q+med			640			
Sondermann [35]	2018	2014	Regional SHI (AOK)	2.8m	any	M2Q	L40.5	PsA	110		No standardisation	
Krüger [36]	2018	2013	Nationwide SHI (InGef)	3.2m	≥18	G, M2Q	M45	AS	322		assumed to be representative	
Steffen [37]	2017	2015	Nationwide SHI (ZI)	60 m	≥15	M2Q+lab	M05, M06	RA	1080 (1060)	80	by age and sex to SHI total population 2016	660,000
Hense [38]	2016	2013	Nationwide SHI (BARMER)	7.1 m	≥18	G, M2Q	M05, M06	RA	1620 (1380)		by age and sex to German standard population 2013	
						G, M2Q+lab			1110 (950)			
						G, M2Q+med			940 (810)			
						G, M2Q+rheumatologist visit			640 (550)			

AAV, anti-neutrophil cytoplasmic antibody-associated vasculitis; AID, autoimmune disease; AS, ankylosing spondylitis; axSpA, axial spondyloarthritis; BD, Behçet disease; CTD, connective tissue disease; DM, dermatomyositis; G (gesichert), confirmed diagnosis; GCA, giant cell arteritis; GPA, granulomatosis with polyangiitis; IIM, idiopathic inflammatory myopathy; iRMD, inflammatory rheumatic disease; JIA, juvenile idiopathic arthritis; lab, laboratory assessment of inflammatory markers; m, million; M2Q, at least 2 diagnoses in 2 quarters or 1 inpatient code; med, prescription of antirheumatic therapy; MPA, microscopic polyangiitis; n.r., not reported; organ, organ involvement; PM, polymyositis; PMR, polymyalgia rheumatica; PsA, psoriatic arthritis; pSjD, primary Sjögren’s disease; RA, rheumatoid arthritis; SHI, statutory health insurance; SjD, Sjögren’s disease; SLE, systemic lupus erythematosus; specialist, specialist care; SSc, systemic sclerosis.

Prevalences are reported for the most recent study year, raw data with 95% confidence intervals, and standardized data in bold.

⁎ The publication was released during the peer-review process. The data had been included from the corresponding conference abstract, published in 2024.

All studies used statutory health insurance (SHI) data—17 nationwide and 3 regional databases. Good representation of the national population was met by the data source from the Central Research Institute of Ambulatory Health Care (ZI) [21,22] that includes all persons with SHI in Germany, corresponding to 88% of the population. The other studies used SHI data with smaller sample sizes from individual health insurance funds (AOK [23], BARMER [27,33,38], BKK [29], TK [26]) or from institutions that process data from ≥1 health insurance companies for analysis purposes (InGef [17,18,24,31,36], Vilua [30], WIG2 [20]). In all studies, iRMDs were identified using ICD-10-GM codes with a confirmed diagnosis. The M2Q criterion, requiring ≥1 inpatient or 2 outpatient diagnoses in 2 quarters within a 12-month period, was applied in 18 studies. All studies reported 1-year prevalences, and 10 studies provided age- and sex-standardized values to the German population. In the overall assessment, 11 studies had a moderate and nine had a high risk of bias (Supplementary Table S1). A high risk of bias was assessed if only ICD codes were considered for the case definition.

Prevalence and incidence data by iRMD are reported in Supplementary Table S2. Searches for data from other European countries yielded 72 studies, reported by iRMD in Supplementary Table S3.

### Rheumatoid arthritis

Reported prevalences for RA range from 0.5% to 0.9% in other European countries [[39], [40], [41], [42], [43], [44], [45]] and from 0.4% to 1.4% in German studies [[22], [23], [24],^32^,^34^,^37^,^38^] (Fig 2). More recent German data report the highest prevalences, with 1.2% to 1.4% in the total population [[21], [22], [23], [24]]. They are consistent with previous studies that exclusively used ICD-10 codes (M05, M06) [30,38] but considerably higher compared with studies requiring more comprehensive case definitions, such as additional examination of laboratory markers. These result in prevalences between 0.95% to 1.1% [37,38], consistent with epidemiological studies [43] and UK administrative data [40]. The German SHI data overestimate RA prevalence and incidence because they include many nonspecific codes (M06.9) [46] and double coding with other iRMDs, such as connective tissue diseases (CTDs), PsA, and SpA in >10% [25]. All SHI data underestimate the epidemiological prevalence because undiagnosed cases and people without medical care are not included. Taking this into account, we estimate the RA prevalence at 1.0%, in line with the only former population-based survey in Germany [47] and our previous assumption, corresponding to ≈700,000 adults with RA. Estimates are based on 69.6 million adults in the German population in 2024 [15] (Table 2*)*.

**Figure 2:**
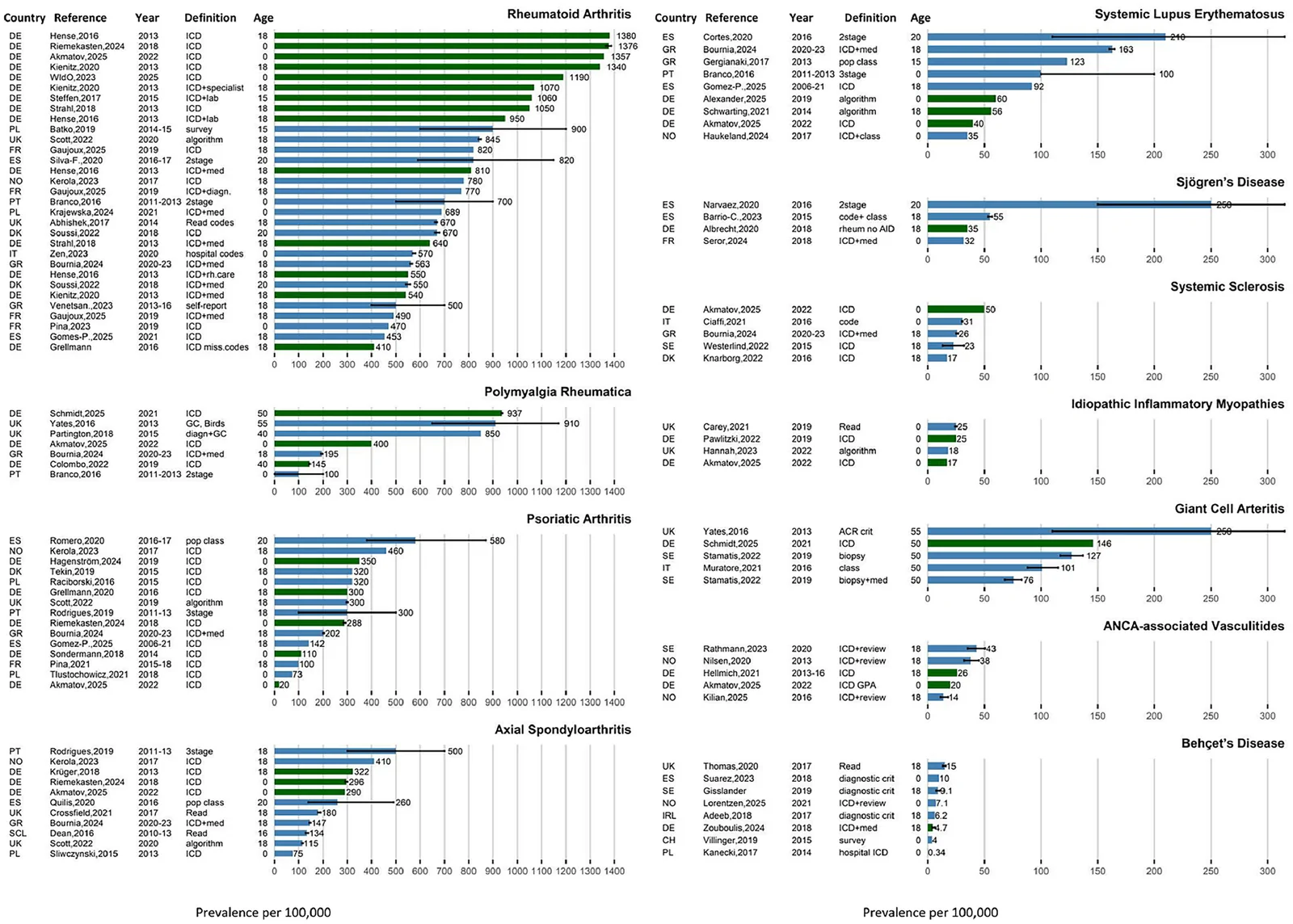
Prevalence data from 2013 onwards, from Germany (green) and other European countries (blue, with official country codes) by inflammatory rheumatic diseases. Shown are first author with year of publication, study year(s), case definition, and minimum age of inclusion. Diagnoses were defined by codes (ICD, Read). ACR, American College of Rheumatology; AID, autoimmune disease; class, classification; crit, criteria; diagn, diagnostic measures; GC, glucocorticoids; Birds, bird criteria; ICD, International Classification of Diseases; lab, laboratory markers; med, medication; miss, missing; pop, population-based study; rh. care, rheumatological care; specialist, specialist diagnosis.

**Table 2:** Estimates on prevalence and incidence of iRMD in Germany

	Prevalence per 100,000					Incidence per 100,000				
	Range in German studies	Range in European studies	Estimate^a^	Estimated number of adults affected^b^	Author’s assessment of reliability	Range in German studies	Range in European studies	Estimate^a^	Estimated number of newly affected adults per year^b^	Author’s assessment of reliability
**Rheumatoid arthritis**	410-1380	470-900	1000	700,000	moderate	40-130	15-94	60	42,000	moderate
**Spondyloarthritis group**	n.a.	1600	1000	700,000	low		14.3	47	33,000	low
Axial spondyloarthritis	290-322	75-500	300	210,000	moderate	40	4.1-11.8	10	7000	low
Psoriatic arthritis	110-350	73-580	300	210,000	low	70	8.4-25.9	17	12,000	low
Peripheral spondyloarthritides^c^	n.a.	n.a.	400	280,000	low			20	14,000	low
**Connective tissue diseases group**	n.a.	n.a.	195	136,000	low			21	15,800	low
Primary Sjögren’s disease	35	32-250	70	49,000	low	4.2	0.7-10.3	8	5600	low
Systemic lupus erythematosus	40-60	35-210	56	39,000	moderate	8.8-8.9	1.8-9.4	8.8	6100	moderate
Systemic sclerosis	50	17-31	35	24,000	low	n.a.	1.2-2.9	4	2700	low
Idiopathic inflammatory myopathies	17-24	14-25	17	12,000	low	2.5 (DM) 1.5 (PM)	0.8-1.7	1	700	low
Mixed connective tissue diseases	n.a.	n.a.	17	12,000	low			1	700	low
**Polymyalgia rheumatica**	153-937^d^	100-910^d^	600 ≥50 y 320 ≥18 y	220,000	low	18-111^d^	27-91	90 ≥50 y 48 ≥18 y	34,000	low
**Vasculitides group**	n.a.	n.a.	115	79,000				15.1	10,500	
Giant cell arteritis	146 ≥50 yrs	76-250	146 ≥50 y 80 ≥18 y	55,000	low	23.6	7.4-22.2	21 ≥50 y 11 ≥18 y	7900	low
ANCA-associated vasculitides	20 (GPA)- 25.6	14-43	26	18,000	moderate	4.6	1.6-3.0	3	2100	low
Behçet disease	4.7	0.3-14.6	4.7	3300	low	0.6	0.29-0.61	0.6	400	low
Other vasculitides	n.a.	n.a.	4.0	2800	low	n.a.	n.a.	0.5	350	low
**Any iRMD**			**2630**	**1,835,000**	**low**			**191**	**130,000**	**low**

ANCA, anti-neutrophil cytoplasmic antibody; DM, dermatomyositis; iRMD, inflammatory rheumatic disease, n.a. not available; PM, polymyositis.

a Based on qualitative assessment of studies.

b Based on 69.6 million adults and 37.4 million people aged ≥50 years in the German population in 2024.

c Enteropathic, reactive, and undifferentiated forms.

d Based on different age groups.

The annual incidence of RA is from 15 to 94 in 100,000 in other European countries [5,[39], [40], [41], [42],[48], [49], [50], [51], [52], [53], [54]] and from 40 to 130 in German studies [32,34,37] (Fig 3). Two of the reported incidences [34,37] are based on stricter case definitions and are within the range of European data. We estimate the annual incidence of RA in Germany to be 60 in 100,000 adults.

**Figure 3:**
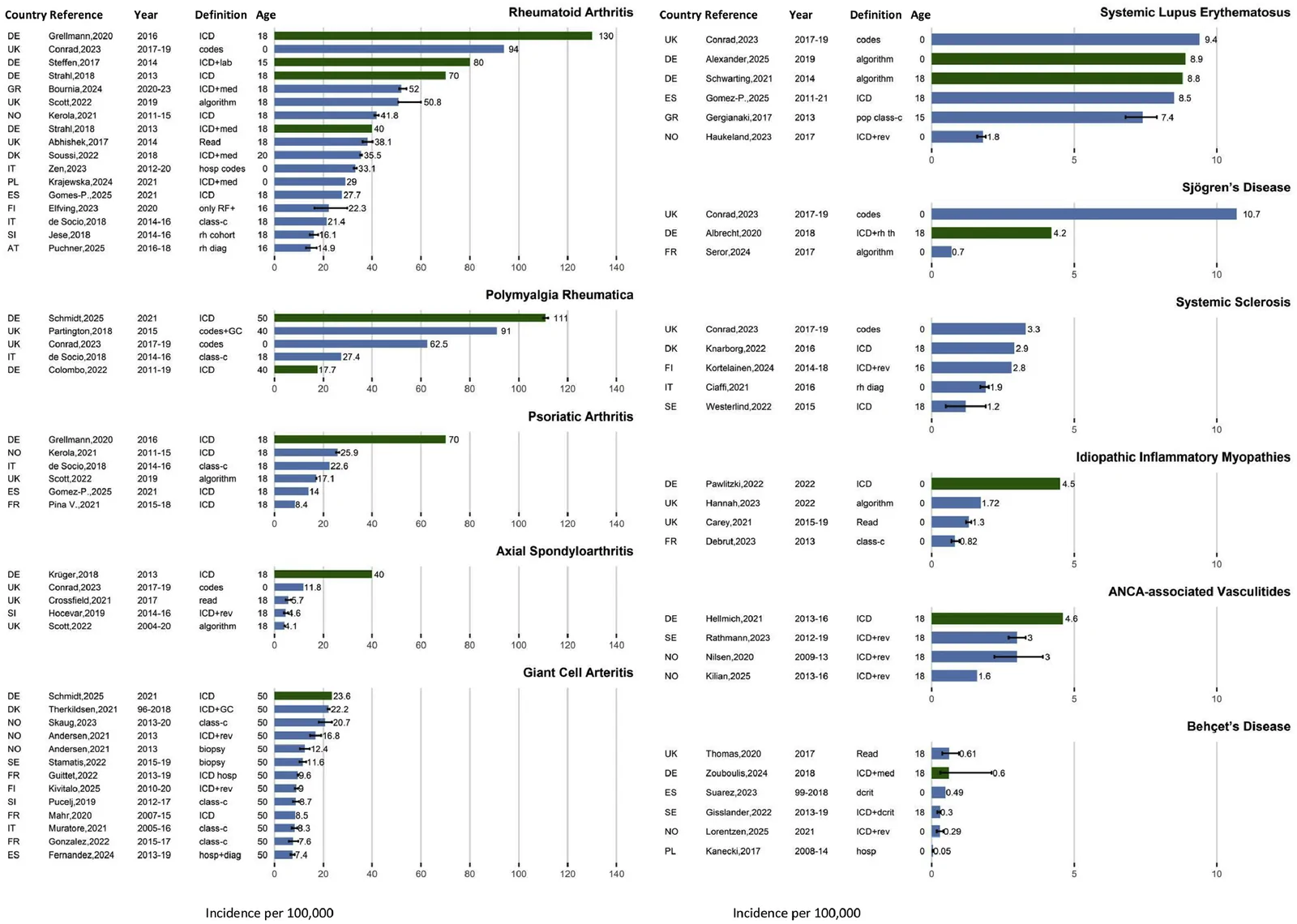
Incidence data from 2013 onwards, from Germany (green) and other European countries (blue, with official country codes) by inflammatory rheumatic diseases. Shown are first author with year of publication, study year(s), case definition and minimum age of inclusion. Diagnoses were defined by codes (ICD, Read). class-c, classification criteria; dcrit, diagnostic criteria; diag, diagnosis; hosp, hospital; lab, laboratory markers; med, medication; pop, population-based study; rev, review; RF, rheumatoid factor; rh diag, rheumatologist diagnosis; rh cohort, rheumatology cohort; rh th, rheumatology therapy

### Axial spondyloarthritis

European axSpA/ankylosing spondylitis (AS) prevalences range from 0.075% for treated AS in Poland to 0.5% for AS in the multistage population-based EpiRheumaPt survey in Portugal [40,44,[55], [56], [57], [58], [59], [60]]. The reported prevalences from Germany are between 0.3% (M45, M46, any age in 2018) and 0.32% (M45, adults in 2013) [24,25,36]. Because the German data are consistent and within the European range, we estimate the prevalence of axSpA to be 0.3%, corresponding to 210,000 adults with axSpA.

The only reported annual incidence of axSpA in Germany is 40 in 100,000, which is too high given the prevalence of 0.3% [36] and the decades-long time lived with disease. AxSpA/AS incidence in the UK ranges from 4.1 to 11.8 in 100,000 [5,40,59]. Because axSpA prevalence in German data is higher than that in the UK, we estimate the annual incidence to be 10 in 100,000.

### Psoriatic arthritis

European data of PsA prevalence range from 73 to 580 per 100,000, with different age and case definitions [40,44,56,[60], [61], [62], [63], [64], [65], [66]]. In 5 German studies, the prevalence of PsA ICD codes (L40.5, M07.0-3) varied from 110 (L40.5 only) [35] to 350 (including juvenile PsA, M09.0) [26] per 100,000 adults [32] or inhabitants of any age, respectively [24]. A meta-analysis of the global prevalence showed that prevalence rates derived from administrative data are slightly lower than those from population-based studies [14]. Based on the German data, we estimate the PsA prevalence to be 0.3%, corresponding to 210,000 adults.

European incidences of PsA range from 8.4 to 25.9 in 100,000 [40,50,54,62,65]. The only reported incidence of PsA in Germany is 70 in 100,000, which is too high, given the prevalence of 0.3% [32], and is far outside the European range. With comparable prevalence to Germany, UK data from Scott et al. [40] reports an incidence of 17 in 100,000 with a reasonable case definition, which we take as the estimate for Germany.

### Spondyloarthritides

The entire group of SpA includes axSpA and peripheral (p)SpA, encompassing PsA, reactive SpA, enteropathic SpA, and undifferentiated forms. The only reported prevalence for SpA is from a population-based study from Portugal, with 1.6% for total SpA, thereof 0.5% AS and 0.3% PsA [60]. No German data are available. A global review indicates that the relative proportion of pSpA among the entire SpA group in cohort studies varies between 10% and 28%, depending on how the overlap between SpA entities is handled [67]. Based on this, it is very difficult to make an estimate for the entire SpA group. We summed up the prevalence of PsA and axSpA and added an estimate of 0.4% for other SpA forms, resulting in an estimated prevalence of 1.0% for SpA and 700,000 adults, comparable to the frequency of RA. A comparable magnitude to RA was also assumed in a former review [68].

By adding the incidence rates for axSpA and PsA and estimating 20 in 100,000 for all other forms of pSpA, we estimate the incidence for SpA to be 47 in 100,000.

### Primary Sjögren’s disease

European prevalences of pSjD are between 32 per 100,000 (treated) inhabitants in French health administrative data [69], 55 per 100,000 adults (fulfilling classification criteria) in a Madrid registry [70], and 250 per 100,000 in the epidemiological EPISER study from Spain, with 50% previously undiagnosed cases [71]. The ZI reports a prevalence of 330 per 100,000 individuals based on the ICD-10 code M35.0 in 2022 [22]. The ICD does not distinguish between primary and associated forms; further Sicca symptoms in diseases other than AIDs are also included. Therefore, these data significantly overestimate the prevalence of pSJD. In the only German study specifying data on pSjD, prevalence of pSjD was 35 per 100,000 when the data was restricted to persons in rheumatology care and without other AIDs [33]. This conforms to the French data, but undiagnosed and untreated patients need to be added. Given the many undiagnosed cases in Spain, we estimate the prevalence of pSjD to be higher, at 70 per 100,000, corresponding to 49,000 adults.

European incidences for pSjD are between 0.7 (algorithm to identify primary SjD) from France [69] and 10.7 (ICD/Read/Snomed codes) from the UK [5]. The German study reports an incidence of 4.2 in 100,000 (rheumatologically treated) adults [33]. Taking into account undiagnosed and non-rheumatologically treated cases, we estimate the incidence of pSjD to be 8 in 100,000 (including undiagnosed and untreated adults).

### Systemic lupus erythematosus

European prevalences of SLE range from 35 to 210 in 100,000 [44,[72], [73], [74], [75], [76]] and German prevalences are between 40 and 60 in 100,000, all with different case and age definitions [20,22,29]. Applying strict inclusion criteria, our previous estimate, which was based on the study of Schwarting et al. [29], remains unchanged with an estimated prevalence of 56 in 100,000, corresponding to 39,000 adults.

Reported incidences of SLE are 1.8 to 9.4 in 100,000 in European [5,72,73,76] and 8.8 to 8.9 in 100,000 in German studies [20,29]. The lowest incidence rates are reported by Haukeland et al. [73], confirming that incidence is overestimated when only ICD codes are considered. As the German studies both considered additional criteria for the case definition, we take their rates to estimate the incidence of SLE at 8.8 in 100,000.

### Systemic sclerosis

European prevalences of SSc range from 17 to 31 per 100,000 [44,[77], [78], [79]]. The only German study from ZI reported a prevalence of 50 per 100,000 inhabitants for the SSc ICD code M34, far outside the European range. It can be assumed that the exclusive use of the ICD code considerably overestimates the prevalence of SSc, overlapping with other CTDs. We took the average of the German and European figures, resulting in a mean value of 35 in 100,000, corresponding to 24,000 adults.

European data on SSc incidence ranges between 1.2 and 2.9 in 100,000 [77,79,80]. Based on the prevalence, we estimated the incidence of SSc correspondingly at 4 in 100,000 adults.

### Idiopathic inflammatory myopathies

European prevalences of IIMs are between 14 and 25 per 100,000 [[81], [82], [83]]. A study with BARMER data reported prevalences in 2019 of 11.3 per 100,000 inhabitants for dermatomyositis (DM), 12.5 for polymyositis (PM), 1.1 for interstitial myositis, and 39.7 for other myositides [27]. The ZI reported a prevalence for DM/PM of 17 per 100,000 inhabitants in 2022 [22]. Both are based on ICD codes and fall within the European range. Because DM and PM codes may overlap, we selected the ZI data as basis for our estimate of 17 in 100,000, corresponding to 12,000 adults.

European incidences of IIMs range from 0.8 to 1.7 in 100,000 [82], [83], [84]. The only German study reported an incidence of 1.5 (PM) and 2.5 (DM) per 100,000 inhabitants in the BARMER data [27]. The lowest incidence was observed in a Capture-Recapture Survey from France, showing that 43% of patients with hospital administrative claims of incident IIM did not match 2017 EULAR/American College of Rheumatology criteria after review [84]. For this reason, we estimate the incidence of IIM to be in the lower range of 1 in 100,000 adults.

### Connective tissue diseases

Adding pSjD, SLE, SSc, IIMs, and an assumption of the prevalence of mixed forms comparable to IIMs, we estimate the total number of CTDs to be 0.2%, corresponding to 136,000 adults.

### Polymyalgia rheumatica

European prevalence studies, 2 of them population-based, range from 100 (any age) in Portugal to 910 in 100,000 individuals aged ≥55 years in the UK [44,74,85,86]. German data are reported from ZI with 400 in 100,000 inhabitants in 2022 for PMR and/or GCA [22] and InGef with 937 per 100,000 individuals aged ≥50 years in 2021 [18]. Both exceed previous data from a regional AOK study of 145 in 100,000 individuals aged ≥40 years [28]. All 3 studies are based on ICD-10 codes M35.3/M31.5 only. Of those persons diagnosed with PMR, only 56% received glucocorticoid (GC) or disease-modifying antirheumatic drug (DMARD) therapy in the InGef study [18], questioning the diagnosis. For those with antirheumatic therapies, misclassification with late-onset RA needs to be considered. In unpublished BARMER data from 2023, 26% of 54,000 individuals with PMR also had an ICD code for RA. For both reasons, we consider the prevalence of PMR based solely on ICD codes to be too high. We take the data from ZI and InGef as a basis and deduct 30% due to the uncertainty of the diagnoses, estimating the prevalence of PMR to be 600 per 100,000 persons aged ≥50 years. This corresponds to 220,000 adults and is notably higher than previously assumed.

Incident PMR data from Europe is reported from Italy (27.4 in 100,000 adults) [50] and 2 studies from the UK (63 per 100,000 inhabitants [5] and 91 per 100,000 aged >40 years [86]). In the German InGef study, the incidence was 111 in 100,000 persons aged ≥50 years in 2021 [18], exceeding the incidence of 17.7 in 100,000 individuals aged >40 years from AOK data [28]. Of those persons with incident ICD codes in the InGef data, 11% had not received any GC or DMARD therapy within the 2 years after assignment of the ICD code. Thus, the diagnosis must also be questioned here. Based on the InGef data, minus diagnostic uncertainty, we estimate the incidence of PMR in Germany to be 90 in 100,000 in those aged ≥50 years.

### Giant cell arteritis

European prevalences of GCA range from 76 in 100,000 for biopsy-proven, treated GCA in Sweden [87] to 250 in 100,000 for adults aged ≥55 years in a population-based study from the UK [9]. German data are available from InGef [19] with 146 in 100,000 in adults aged ≥50 years. This is 3 times higher compared with a former regional survey from 2006 [88]. We take the prevalence from InGef as estimate, corresponding to 80 per 100,000 in the adult population or 55,000 people. This is significantly higher than previously estimated.

European incidences of GCA range from 7.4 to 22.2 per 100,000 [86,[89], [90], [91], [92], [93], [94], [95], [96], [97], [98]]. A north-south gradient has been described for the incidence of GCA, being highest in Scandinavia [99]. In the InGef data, GCA incidence was 23.6 per 100,000 persons aged ≥50 years. This is the highest reported incidence in Europe but refers to ICD codes only. As the studies from Norway (20.7) [96] and Denmark (22.2) [89] considered GC therapy or classification criteria for case validation, we take these as a more reliable basis for our estimate, resulting in an estimated incidence of 21 in 100,000 in those aged ≥50 years.

### ANCA-associated vasculitides

The prevalence of AAV in total ranges from 14.4 to 42.8 per 100,000 adults in Northern Europe [[100], [101], [102]]. For granulomatosis with polyangiitis (GPA), the ZI reports a prevalence of 20 per 100,000 population in 2022 [22], comparable to 21 in 100,000 adults for GPA reported by InGef in 2016 [31], and 4.6 in 100,000 for microscopic polyangiitis (MPA). Our estimate remains at 26 in 100,000 adults based on the InGef study, corresponding to 18,000 adults.

Incidence of AAV ranges from 1.6 to 3 in 100,000 in Northern Europe [[100], [101], [102]] and was highest with 4.6 in 100,000 in the German InGef data. A chart review performed by Kilian et al. [100] confirmed a clinical diagnosis of AAV in only 60% of the cases registered at least once with an AAV-specific ICD-10 code. Therefore, we estimate the incidence lower than reported by InGef but within the European range at 3 in 100,000 adults.

### Behçet Disease

European prevalences of BD range from 0.3 to 14.6 per 100,000 [[103], [104], [105], [106], [107], [108], [109]], depending on the case definitions used. Zouboulis et al. [17] reported a prevalence of 4.7 and an incidence of 0.6 per 100,000 adults in 2018 using the InGef database. Two population-based studies from Ireland and Switzerland [103,108] are consistent with the data from Zouboulis et al. We therefore adopt an estimate of 4.7, which corresponds to 3300 adults with BD and 400 incident patients per year.

### Takayasu arteriitis

European prevalence data of Takayasu arteriitis (TAK) ranges from 0.46 to 3.3 in 100,000 [94,[110], [111], [112]] and incidence rates from 0.03 to 0.09 in 100,000 [110,111]. No data are available from Germany.

### Vasculitides

The total estimated number of adults with GCA, AAV, and BD is 76,000. To this number, approximately 1400 adults with TAK and a comparable number of adults with other rare forms of vasculitis can be added. This gives us an estimated prevalence for the entire group of vasculitides of 115 in 100,000 adults and approximately 79,000 adults with a vasculitis.

### Total iRMDs

The estimated prevalence of iRMDs in Germany is 2.6%, corresponding to 1.8 million adults affected, when the prevalences of RA, SpA, CTDs, and vasculitides are added together. Summing up the incidences of all iRMDs results in an incidence of 0.2%, corresponding to 130,000 adults newly affected per year (Fig 4).

**Figure 4:**
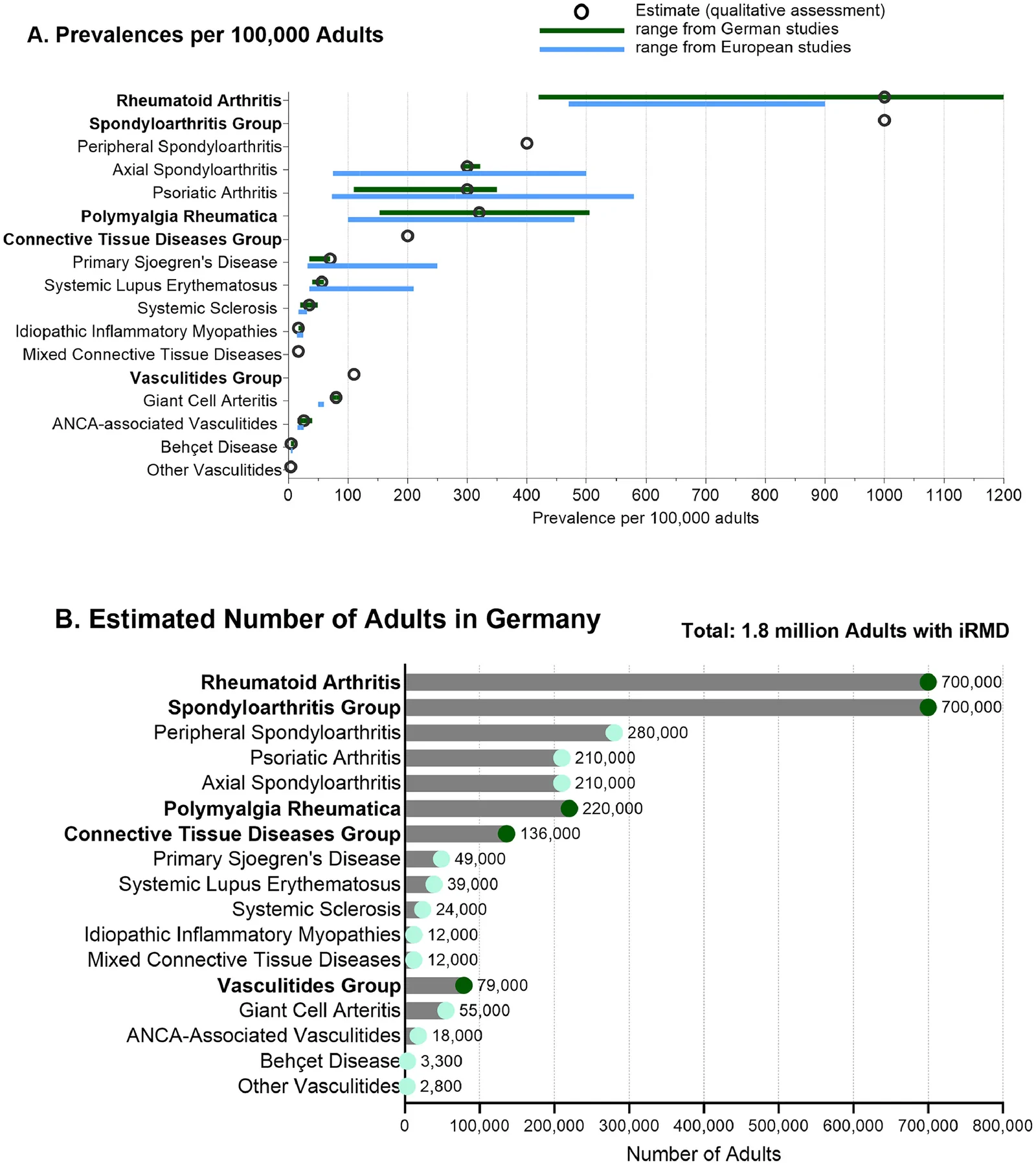
Prevalence ranges (A) and estimated number of adults (B) with inflammatory rheumatic disease (iRMD) in Germany.

## DISCUSSION

This work provides an up-to-date estimate on the number of adults living with iRMDs in Germany. It is based on German original studies with prevalence and incidence data from 2013 to the present and on comparison with data from other European countries. Updated calculations confirm our previous prevalence estimate of approximately 1.8 million adults with iRMDs in Germany and add an estimate for the incidence of around 130,000 newly affected adults per year. These reference figures are essential for specialist training, workforce, and capacity planning, particularly in times of limited rheumatology specialist resources [3]. The systematic review yielded numerous new studies, leading to adjustments of our previous estimates for individual iRMDs [4] while the total number of people affected remained changed based on the 2024 population figures. For the first time, we estimated the overall annual incidence of iRMDs in Germany. This figure is relevant for capacity planning of new patient appointments, which require longer initial rheumatology consultations and closer follow-up during the first year after diagnosis.

Comparison of German data with those from other European countries shows significant deviations. The majority of the variance can be explained by the heterogeneous methodology of the studies. The exclusive use of ICD-based case definitions often resulted in higher prevalence and incidence estimates in German studies compared with other European studies using stricter definitions or multistage population-based methods. A qualitative assessment of the data obtained from heterogeneous study designs was essential to provide reliable information for future rheumatology workforce planning. Although the calculation focused on German data, the methodological approach is applicable to other countries, and comprehensive evidence on the prevalence and incidence of 11 iRMDs across Europe is provided.

Data from the Global Burden of Disease Study (GBD) are also used for prevalence estimates [6]. In this data, the age-standardized RA prevalence for Germany is estimated to be significantly lower, ranging from 0.2% to 0.4% [6,113]. The GBD uses a world standard population that has a substantially different age distribution than that of the German population. The RA prevalence reported from ZI [37] would change from 1.2% to 0.6% using the GBD world standard population. The reported prevalence for Germany from the GBD was calculated via a meta-regression model using prevalence data from comparable countries but no primary sources from Germany. This is why we refrain from using the GBD prevalence estimates for Germany.

### Strengths and limitations

Since the last systematic review, data quality has improved considerably, as all newly included studies used nationwide data and considered at least 2 outpatient diagnoses for case definition. The data from the ZI are particularly important as they represent the majority of the German population [21,22].

A major limitation of the provided results is the exclusive use of claims data in German prevalence and incidence studies. Administrative data are collected for reimbursement purposes, which limits data validity. Several studies show that ICD-coded iRMD diagnoses cannot be confirmed by classification criteria in a substantial proportion of cases [73,84,100]. Within German SHI data, up to 10% of individuals have more than one iRMD coded [22], leading to overestimations. On the other hand, a relevant number of undiagnosed cases are missed within SHI databases, resulting in underestimation of the epidemiological prevalence. These can only be identified with multistage population-based methodology [71], which is much more resource-intensive and time-consuming and therefore is rarely performed. Prevalence and incidence estimates are also sensitive to the number of administrative data years examined: short time period analyses result in lower prevalence estimates, undercounting individuals who are not recorded within a short time window while incidence is higher, overcounting prevalent cases as new diagnoses [114]. By qualitative assessment, we considered methodological pitfalls in administrative prevalence studies, including cohort assembling, validity of case ascertainment, and counting the number affected [115]. To account for diagnosis-specific differences in ICD-10 code reliability, we analysed each iRMD separately. Coding quality varies between countries, and the German coding quality is rather poor with excessive use of unspecific x.9 or M06 codes [46].

## Conclusion

This systematic review on the prevalence and incidence of iRMDs provides current numbers on people affected by iRMD. We estimate that in 2024, about 1.8 million adults in Germany lived with iRMDs. with approximately 130,000 incident cases per year. All German studies included are based on claims data with a moderate to high risk of bias. The comparison with other European studies using different methodologies proved helpful for prevalence and incidence assessments. Our study provides, for the first time, compiled European epidemiological data on 11 iRMDs. The methodology sets a blueprint on how to provide estimates for the different inflammatory diseases in European countries. These data are key to provide up-to-date numbers on people living with iRMDs in Europe and can serve as a hallmark for the RheumaFacts study.

## Editor disclosure

The peer review process did not involve Editorial Board Member Anja Strangfeld, and the editorial decision-making was led by editors who were not involved in the creation of this manuscript.

## Competing interests

Anja Strangfeld reports a relationship with European Alliance of Associations for Rheumatology (EULAR) that includes: board membership. Katinka Albrecht, Anja Strangfeld, Johanna Callhoff reports a relationship with German Society for Rheumatology (DGRh) that includes: board membership. Anja Strangfeld reports a relationship with AbbVie, Pfizer, Galapagos, AlfaSigma, Takeda, Janssen, Lilly, and UCB that includes: speaking and lecture fees. Johanna Callhoff reports a relationship with Janssen, Pfizer, Idorsia that includes: speaking and lecture fees. If there are other authors, they declare that they have no known competing financial interests or personal relationships that could have appeared to influence the work reported in this paper.

## Appendix Supplementary materials

Image, application 1

## Footnotes

Supplementary material associated with this article can be found in the online version at doi:10.1016/j.ero.2026.100208.
