# Supplementary material for: The epidemiology of inflammatory rheumatic diseases in Germany in a European context: a systematic review to support workforce planning
Source: EULAR Rheumatol Open. 2026 Jul 9;2(3):100208. doi: 10.1016/j.ero.2026.100208 (PMC13650311; doi:10.1016/j.ero.2026.100208)
Supplement: Supplementary file 1 [file mmc1.docx]

Supplementary

Search terms for PubMed, last search on 3 November 2025

| #1 | prevalence[Title/Abstract] OR incidence[Title/Abstract] | 1,892,429 |
| --- | --- | --- |
| #2 | rheumatoid arthritis[Title/Abstract] OR rheumatic disease[Title/Abstract] OR ankylosing spondylitis[Title/Abstract] OR spondyloarthritis[Title/Abstract] OR psoriatic arthritis[Title/Abstract] OR systemic lupus[Title/Abstract] OR Sjögren*[Title/Abstract] OR Sjoegren*[Title/abstract] OR systemic sclerosis[Title/Abstract] OR myositis[Title/Abstract] OR inflammatory myopathies[Title/Abstract] OR polymyalgia rheumatica[Title/Abstract] OR ANCA-associated vasculiti*[Title/Abstract] OR giant cell arteritis[Title/Abstract] OR polymyalgia rheumatica[Title/Abstract] OR Behcet's disease[Title/Abstract] | 293,182 |
| #3 | German*[Title/Abstract] | 194,949 |
|  | #1 AND #2 AND #3 | 241 |
|  | Publication date 01/01/2015 to 03/11/2025 | 150 |

Web of Science search on 27 November 2025

(PY=(2015-2025)) AND (SO=(prevalence OR incidence) OR AB=(prevalence OR incidence)) AND (SO=(German*) OR AB=(German*)) AND (SO=(rheumatoid arthritis OR rheumatic disease OR ankylosing spondylitis OR spondyloarthritis OR psoriatic arthritis OR systemic lupus OR Sjögren* OR Sjoegren* OR systemic sclerosis OR myositis OR inflammatory myopathies OR polymyalgia rheumatica OR ANCA-associated vasculiti* OR giant cell arteritis OR polymyalgia rheumatica OR Behcet's disease) OR AB=(rheumatoid arthritis OR rheumatic disease OR ankylosing spondylitis OR spondyloarthritis OR psoriatic arthritis OR systemic lupus OR Sjögren* OR Sjoegren* OR systemic sclerosis OR myositis OR inflammatory myopathies OR polymyalgia rheumatica OR ANCA-associated vasculiti* OR giant cell arteritis OR polymyalgia rheumatica OR Behcet's disease))

Suppl Table 1 Risk of bias assessment

|  | 1. Was the target population of the study a good representation of the national population in terms of the relevant variables? | 2. Was the sampling frame a true or accurate representation of the target population? | 3. Was a form of random selection used to select the sample OR was a census conducted? | 4. Was the probability of non-response bias minimal? | 5. Was the data collected directly from the subjects (as opposed to a proxy)? | 6. Did the study use an acceptable case definition? | 7. Was the study instrument used to measure the parameter of interest valid and reliable? | 8. Was the same type of data collection used for all subjects? | 9. Was the length of the shortest prevalence period appropriate for the parameter of interest? | 10. Were the numerator(s) and denominator(s) appropriate for the parameter of interest? | 11. Summary item on overall risk of study bias |
| --- | --- | --- | --- | --- | --- | --- | --- | --- | --- | --- | --- |
| Zouboulis 2025 | low | low | low | low | high | low | high | low | low | low | moderate |
| Schmidt 2025 | low | low | low | low | high | high | high | low | low | low | high |
| Alexander 2025 | low | low | low | low | high | low | high | low | low | low | moderate |
| WIdO 2025 | high | low | low | low | high | high | high | low | low | low | high |
| Akmatov 2024,25 | low | low | low | low | high | high | high | low | low | low | moderate |
| Hagenström 2024 | high | low | low | low | high | low | high | low | low | low | moderate |
| Riemekasten 2024 | low | low | low | low | high | high | high | low | low | low | high |
| Pawlitzki 2022 | high | low | low | low | high | high | high | low | low | low | high |
| Included in Systematic review from 2022 | | | | | | | | | | | |
| Colombo 2022 | high | high | low | low | n.a. | high | high | low | low | low | high |
| Schwarting 2021 | high | low | low | low | n.a. | low | high | low | low | low | moderate |
| Hellmich 2021 | low | low | low | low | n.a. | low | high | low | low | low | moderate |
| Kienitz 2020 | low | low | low | low | n.a. | low | high | low | low | low | moderate |
| Grellmann 2020 | low | low | low | low | n.a. | high | high | low | low | high | moderate |
| Albrecht 2020 | high | low | low | low | n.a. | high | high | low | low | low | high |
| Sondermann 2018 | high | high | low | low | n.a. | high | high | low | high | high | high |
| Krüger 2018 | low | low | low | low | n.a. | low | high | low | low | low | moderate |
| Strahl 2018 | high | high | low | low | n.a. | low | high | low | low | low | high |
| Steffen 2017 | low | low | low | low | n.a. | low | high | low | low | low | moderate |
| Hense 2016 | high | low | low | low | n.a. | low | high | low | low | low | moderate |
| Two-stage assessment of the individual criteria: low or high, three-stage overall assessment: low, moderate or high. N.a. not applicable  Checklist according to Hoy D, Brooks P, Woolf A, Blyth F, March L, Bain C, et al. Assessing risk of bias in prevalence studies: modification of an existing tool and evidence of interrater agreement. J Clin Epidemiol. 2012;65(9):934–9. | | | | | | | | | | | |

Suppl Table 2 Prevalence and incidence data from German studies from 2013 onwards by iRMD

| iRMD | Reference | most recent Study Year | Data source | Distribution | Sample size | Age | Case definition | ICD Codes | Prevalence  per 100,000  (95% CI) | Incidence  per 100,000 (95% CI) |
| --- | --- | --- | --- | --- | --- | --- | --- | --- | --- | --- |
| RA | Akmatov 2025 | 2022 | SHI (ZI) | national | 73m | any | G, M2Q | M05, M06 | 1300 |  |
|  | WIdO 2025 | 2023 | SHI (AOK) | national | 27m | any | G, M2Q | M05, M06 | 1190 |  |
|  | Riemekasten 2024 | 2018 | SHI (InGef) | national | 4m | any | G, M2Q | M05, M06 | 1376 (1365-1387) |  |
|  | Grellmann 2020 | 2016 | SHI (Vilua) | national | 2m | ≥ 18 | n.r. | M05.8, M06.0, M06.8 | 410 | 130 |
|  | Steffen 2017 | 2015 | SHI (ZI) | national | 60m | ≥ 15 | G, M2Q+lab | M05, M06 | 1060 | 80 |
|  | Strahl 2018 | 2013 | SHI (AOK) | regional | 3m | ≥ 18 | G, M2Q | M05, M06 | 1050 | 70 |
|  |  |  |  |  |  |  | G, M2Q+med |  | 640 | 40 |
|  | Hense 2016 | 2013 | SHI (Barmer) | national | 7m | ≥ 18 | G, M2Q | M05, M06 | 1380 |  |
|  |  |  |  |  |  |  | +lab |  | 950 |  |
|  |  |  |  |  |  |  | +med |  | 810 |  |
|  |  |  |  |  |  |  | +rheumatologist visit |  | 550 |  |
|  | Kienitz 2020 | 2013 | SHI (Vilua) | national | 2m | ≥ 18 | G, M2Q | M05, M06 | 1340 |  |
|  |  |  |  |  |  |  | G, M2Q+specialist diagnosis |  | 1070 |  |
|  |  |  |  |  |  |  | G, M2Q+med |  | 540 |  |
| axSpA | Akmatov 2025 | 2022 | SHI (ZI) | national | 73m | any | G, M2Q | M45 | 290 |  |
|  | Riemekasten 2024 | 2018 | SHI (InGef) | national | 4m | any | G, M2Q | M45, M46 | 296 (291–302) |  |
|  | Krüger 2018 | 2013 | SHI (InGef) | national | 3m | ≥ 18 | G, M2Q | M45 | 322 | 40 |
| PsA | Akmatov 2025 | 2022 | SHI (ZI) | national | 73m | any | G, M2Q | L40.5, M07.0-3 | 20 |  |
|  | Hagenström 2024 | 2019 | SHI (TK) | national | 11m | any | G, M2Q | L40.5, M07.0-3, M09.0 | 350 (350) |  |
|  | Riemekasten 2024 | 2018 | SHI (InGef) | national | 4m | any | G, M2Q | L40.5, M07 | 288 (283-293) |  |
|  | Grellmann 2020 | 2016 | SHI (Vilua) | national | 2m | ≥ 18 | G | L40.5, M07.0-3 | 300 | 70 |
|  | Sondermann 2018 | 2014 | SHI (AOK) | regional | 3m | any | M2Q | L40.5 | 110 |  |
| SjD | Akmatov 2025 | 2022 | SHI (ZI) | national | 73m | any | G, M2Q | M35.0 | 330 |  |
|  | Albrecht 2020 | 2018 | SHI (Barmer) | national | 7m | ≥ 18 | G, M2Q +rheumatologist, no AID +rheumatologist + AID | M35.0 | 750 35 48 | 100 4.2 4.1 |
| SLE | Alexander 2025 | 2019 | SHI (WIG2) | national | n.r. | any | G, M2Q+lab/med/specialist | M32.1,8,9 | 60 | 8.9 |
|  | Akmatov 2025 | 2022 | SHI (ZI) | national | 73m | any | G, M2Q | M32.1,8,9 | 40 |  |
|  | Schwarting 2021 | 2014 | SHI (BKK) | national | 4m | ≥ 18 | G, M2Q+lab/med/specialist | M32.1,8,9 | 56 | 8.8 |
| SSc | Akmatov 2025 | 2022 | SHI (ZI) | national | 73m | any | G, M2Q |  | 50 |  |
| IIM | Akmatov 2025 | 2022 | SHI (ZI) | national | 73m | any | G, M2Q | DM/PM: M33 | 17 |  |
|  | Pawlitzki 2022 | 2019 | SHI (Barmer) | national | 9m | any | G | DM: M33.0, M33.1, M33.9 | 11.3 | 2.5 |
|  |  |  |  |  |  |  |  | PM: M33.2 | 12.5 | 1.5 |
|  |  |  |  |  |  |  |  | IM: M60.1 | 1.1 |  |
|  |  |  |  |  |  |  |  | other: M60.8, M60.9, G72.4 | 39.7 |  |
| PMR | Schmidt 2025 | 2021 | SHI (InGef) | national | 5m | ≥ 50 | G, M2Q | M35.3, M31.5 | 937 (934-940) | 111 (110-112) |
|  | Akmatov 2025 | 2022 | SHI (ZI) | national | 73m | any | G, M2Q | M35.3, M31.5, M31.6 | 400 |  |
|  | Colombo 2022 | 2019 | SHI (AOK) | regional | n.r. | ≥ 40 | G, M2Q | M35.3, M31.5 | 145 (131-147) | 17.7 |
| GCA | Schmidt 2025 | 2021 | SHI (InGef) | national | 5m | ≥ 50 | G, M2Q | M31.5, M31.6 | 146 | 23.6 |
| AAV | Akmatov 2025 | 2022 | SHI (ZI) | national | 73m | any | G, M2Q | GPA: M31.3 | 20 |  |
|  | Hellmich 2021 | 2016 | SHI (InGef) | national | 3m | ≥ 18 | G, M2Q | GPA: M31.3 | 21 | 3.4 |
|  |  |  |  |  |  |  |  | MPA: M31.7 | 4.6 | 0.4 |
| BD | Zouboulis 2025 | 2018 | SHI (InGef) | national | 4m | ≥ 18 | G, M2Q | M35.2 | 4.7 | 0.6 |
| n.r. not reported, G (gesichert): confirmed diagnosis, M2Q at least one inpatient or two outpatient diagnoses in two quarters of a year, lab: measure of inflammatory markers, med: antiinflammatory therapy | | | | | | | | | | |
| RA Rheumatoid arthritis, axSpA axial Spondyloarthritis, PsA Psoriatic Arthritis, SjD Sjoegren’s Disease, SLE systemic Lupus erythematosus, SSc Systemic Sclerosis, IIM Idiopathic Inflammatory Myopathies, PMR Polymyalgia rheumatica, GCA Giant Cell Arteritis, AAV ANCA-associated Vasculitides, ZI Zentralinstitut für die kassenärztliche Versorgung, InGef Institut für angewandte Gesundheitsforschung, TK Techniker Krankenkasse, BKK Betriebskrankenkasse, Vilua Vilua Healthcare GmbH | | | | | | | | | | |

Suppl Table 3 Prevalence and incidence data from other European countries from 2013 onwards by iRMD

|  | **Country** | **Reference** | **most recent Study Year** | **Data source** | **Age** | **Case Definition** | **Prevalence per 100,000 (95% CI)** | **Time period** | **Incidence per 100,000 (95% CI)** |
| --- | --- | --- | --- | --- | --- | --- | --- | --- | --- |
| Rheumatoid arthritis | Austria | Puchner 2025 | 2018 | pop | ≥16 | rheumatologist diagnosis |  | 2016-18 | 14.9 (12.9-17.3) |
|  | Denmark | Soussi 2022 | 2018 | adm | ≥20 | ICD+med | 600 (500-600) |  | 35.5 (35.1–35.9] |
|  | Finland | Elfving 2023 | 2020 | pop | ≥16 | only +RA, classification criteria |  |  | 22.3 (15.9-28.8) |
|  | France | Gaujoux-Viala 2025 | 2019 | adm | ≥18 | ICD (+med) different algorithms | 490 to 820 |  |  |
|  | France | Pina Vegas 2023 | 2019 | adm | any | ICD | 470 |  |  |
|  | Greece | Bournia 2024 | 2023 | adm | ≥18 | ICD + medication | 563 (559-568) |  | 52 (51-54) |
|  | Greece | Venetsanopoulo 2023 | 2016 | survey | ≥18 | self-report | 500 (400-700) |  |  |
|  | Italy | de Socio 2018 | 2016 | pop | ≥18 | classification criteria |  |  | 21.4 |
|  | Italy | Zen 2023 | 2020 | adm | any | hospital codes | 570 (570-580) |  | 33.1 (32.5-33.7) |
|  | Norway | Kerola 2023 | 2017 | adm | ≥18 | ICD | 780 |  |  |
|  | Norway | Kerola 2021 | 2015 | adm | ≥18 | ICD |  |  | 41.8 (40.9-42.7) |
|  | Poland | Krajewska 2024 | 2021 | adm | any | ICD + medication | 689 |  | 29 |
|  | Poland | Batko 2019 | 2015 | survey | ≥15 | Survey | 900 (600-1200) |  |  |
|  | Portugal | Branco 2016 | 2013 | pop | any | 2 stage | 700 (500-900) |  |  |
|  | Slovenia | Jese 2018 | 2016 | adm | ≥18 | rheumatology cohort |  |  | 16.1 (14.5-17.9) |
|  | Spain | Gomez-Puerta 2025 | 2021 | adm | ≥18 | ICD | 453 |  | 22.2 |
|  | Spain | Silva-Fernandez 2020 | 2017 | survey | ≥20 | physician diagnosed | 820 (590-1150) |  |  |
|  | UK | Conrad 2023 | 2019 | adm | any | ICD/Read/snomed codes |  |  | 94 |
|  | UK | Scott 2022 | 2020 | adm | ≥18 | Read codes + algorithm | 845 (840-841) |  | 50.8 (50.6-60.0) |
|  | UK | Abhishek 2017 | 2014 | adm | ≥18 | Read codes | 670 (660-670) |  | 38.1 (36.1-40.2) |
| Spondyloarthritis | Denmark | Nygaard 2020 | 2013 | adm | ≥18 | ICD AS |  |  | ?? |
|  | Greece | Bournia 2024 | 2023 | adm | ≥18 | ICD + medication AS | 147 (144-149) |  |  |
|  | Norway | Kerola 2023 | 2017 | adm | ≥18 | ICD axSpA | 410 |  |  |
|  | Poland | Sliwczynski 2015 | 2013 | adm | any | ICD treated AS | 74.8 |  |  |
|  | Portugal | Rodriguez 2019 | 2013 | pop | ≥18 | 3 stage | SpA 1600 (1200-2100) AS 500 (300-700) |  |  |
|  | Scotland | Dean 2016 | 2013 | adm | >15 | read AS | 180 (179-190) |  |  |
|  | Slovenia | Hocevar 2019 | 2016 | adm | ≥18 | ICD SpA |  |  | SpA 14.3 (12.8-16.9) axSpA 4.6 (3.8-5.6) |
|  | Spain | Quilis 2020 | 2016 | pop | ≥20 | AS classification criteria | 260 (140-490) |  |  |
|  | UK | Crossfield 2021 | 2017 | adm | ≥18 | read AS | 180 (170-190) |  | 5.7 (4.6-6.8) |
|  | UK | Scott 2022 | 2020 | adm | ≥18 | read+algorithm axspA | 115 (113-117) |  | 4.1 (4.1-4.2) |
|  | UK | Conrad 2023 | 2019 | adm | any | AS ICD/Read/snomed |  |  | 11.8 |
| Psoriatic arthritis | Denmark | Tekin 2019 | 2015 | adm | ≥18 | ICD | 320 |  |  |
|  | France | Pina Vegas 2021 | 2018 | adm | ≥18 | ICD | 100 |  | 8.4 |
|  | Greece | Bournia 2024 | 2023 | adm | ≥18 | ICD+med | 202 (199-205) |  |  |
|  | Italy | de Socio 2018 | 2016 | pop | ≥18 | classification criteria |  |  | 22.6 |
|  | Norway | Kerola 2021 | 2015 | adm | ≥18 | ICD |  |  | 25.9 (25.2-26.6) |
|  | Norway | Kerola 2023 | 2017 | adm | ≥18 | ICD | 460 |  |  |
|  | Poland | Raciborski 2016 | 2015 | adm | any | ICD | 320 |  |  |
|  | Poland | Tlustochowicz 2021 | 2018 | adm | any | ICD | 73.1 |  |  |
|  | Portugal | Rodriguez 2019 | 2013 | pop | ≥18 | classification criteria | 300 (100-500) |  |  |
|  | Spain | Gomez-Puerta 2025 | 2021 | adm | ≥18 | ICD | 142 |  | 10.4 |
|  | Spain | Romero Perez 2020 | 2017 | pop | ≥20 | classification criteria | 580 (380-870) |  |  |
|  | UK | Scott 2022 | 2019 | adm | ≥18 | ICD+algorithm | 300 (296-303) |  | 17.1 (17.1-17.2) |
| Sjogren's Disease | France | Seror 2024 | 2018 | adm | any | ICD+med ICD+med+AID | pSjD 32 sSjD+AID 20 | 2012-17 | pSjD 0.7-4.3 sSjD 0.3-2.0 |
|  | Spain | Barrio-Cortez 2023 | 2015 | adm | ≥18 | ICD+classification criteria | pSjD 55 (53-57) sSjD 28 (27-29) total SjD 84 (82-87) |  |  |
|  | Spain | Narvaez 2020 | 2016 | pop | ≥20 | pSjD classification criteria | pSjD 250 (150–430) total SjD 330 (210-530) |  |  |
|  | UK | Conrad 2023 | 2019 | adm | any | ICD/Read/Snomed codes |  |  | 10.7 |
| Systemic Lupus Erythematosus | Greece | Bournia 2024 | 2023 | adm | ≥18 | ICD | 163 (160-165) |  |  |
|  | Greece | Gergianaki 2017 | 2013 | pop | ≥15 | classification criteria | 123 |  | 7.4 (6.8-7.9) |
|  | Norway | Haukeland 2024 | 2017 | adm | any | ICD + review | 35 |  | 1.8 (1.6-1.9) |
|  | Portugal | Branco 2016 | 2013 | pop | ≥18 | classification criteria | 100 (100-200) |  |  |
|  | Spain | Cortez Verdu 2020 | 2016 | pop | ≥20 | classification criteria | 210 (110-400) |  |  |
|  | Spain | Gomez-Puerta 2025 | 2021 | adm | ≥18 | ICD | 92.3 |  | 8.5 |
|  | UK | Conrad 2023 | 2019 | adm | any | ICD/Read/Snomed codes |  |  | 9.4 |
| Systemic sclerosis | Denmark | Knarborg 2022 | 2016 | adm | ≥18 | ICD | 16.9 |  | 2.9 |
|  | Finland | Kortelainen 2024 | 2018 | adm | ≥16 | ICD + classification criteria |  |  | 2.8 |
|  | Greece | Bournia 2024 | 2023 | adm | ≥18 | ICD + med | 26 (25-27) |  |  |
|  | Italy | Ciaffi 2021 | 2016 | adm | any | co-payment exemption codes | 30.6 (30.1-31.1) |  | 1.9 (1.7-2.0) |
|  | Sweden | Westerlind 2022 | 2015 | adm | ≥18 | ICD | 22.7 (13.3-32.0) |  | 1.2 (0.5-1.9) |
|  | UK | Conrad 2023 | 2019 | adm | any | ICD/Read/Snomed codes |  |  | 3.3 |
| Idiopathic inflammatory myopathies | France | Debrut 2023 | 2013 | pop | any | Classification criteria, recapture |  | 2006-13 | 0.8 (0.9-1.0) |
|  | UK | Carey 2021 | 2019 | adm | any | Read codes | 25 (24.1-25.8) |  | 1.3 (1.2-1.4) |
|  | UK | Hannah 2023 | 2022 | adm | any | ICD algorithm | 17.9 |  | 1.7 |
| Polymyalgia rheumatica | Portugal | Branco 2016 | 2013 | pop | any | 2 stage | 100 (0-200) |  |  |
|  | Greece | Bournia 2024 | 2023 | adm | ≥18 | ICD + medication | 195 (192-198) |  |  |
|  | Italy | de Socio 2018 | 2016 | pop | ≥18 | classification criteria |  |  | 27.4 |
|  | UK | Yates 2016 | 2013 | pop | ≥55 | GC, classification criteria | cum 910 (650-1170) |  |  |
|  | UK | Partington 2018 | 2016 | adm | >40 | Codes + GC | 850 |  | 91 per person years |
|  | UK | Conrad 2023 | 2019 | adm | any | ICD/Read/snomed |  |  | 62.5 |
| Giant cell arteritis | Denmark | Therkildsen 2021 | 2018 | adm | ≥50 | ICD+GC |  |  | 22.2 (21.8-22.7) |
|  | Finland | Kivitalo 2025 | 2020 | adm | ≥50 | ICD + review |  |  | 9.0 (8.3-9.7) |
|  | France | Gonzalez 2022 | 2017 | pop | ≥50 | classification criteria |  |  | 7.6 (5.9–9.8) |
|  | France | Guittet 2022 | 2019 | adm | ≥50 | ICD |  |  | 9.6 (9.5-9.8) (>50) |
|  | France | Mahr 2020 | 2015 | adm | ≥50 | ICD |  |  | 7 to 10 > 50 yrs. |
|  | Italy | Muratore 2021 | 2016 | pop | ≥50 | classification criteria | >50: 101 (88-115) any age: 42 (37-48) |  | 8.3 (7.1, 9.4) |
|  | Norway | Andersen 2021 | 2019 | adm | ≥50 | ICD + review + biopsy |  |  | 12.4-16.8 |
|  | Norway | Skaug 2023 | 2020 | adm | ≥50 | ICD + classification criteria |  |  | 20.7 (18.2–23.5) |
|  | UK | Yates 2016 | 2013 | pop | ≥55 | classification criteria | cum 250 (110-390) |  |  |
|  | Slovenia | Pucelj 2018 | 2017 | pop | ≥50 | classification criteria |  |  | 8.7 (7.5–10.1) >50yrs |
|  | Spain | Fernandez 2024 | 2019 | adm | ≥50 | classification criteria |  |  | 7.4 (6.6-8.3) |
|  | Sweden | Stamatis 2022 | 2019 | adm | ≥50 | biopsy biopsy + medication | 127 (117-137 76 (68-83) |  | 11.6 (10.3-13) |
| ANCA-associated vasculitides | Norway | Kilian 2025 | 2016 | adm | ≥18 | ICD + review | 14.4 (11.3-18.0) | 2013-16 | 1.6 |
|  | Norway | Nielsen 2020 | 2013 | adm | ≥18 | ICD + review | 38.3 (32.2-45.2) | 2009-13 | 3.0 (2.2-3.9) |
|  | Sweden | Rathmann 2023 | 2019 | adm | ≥18 | ICD + review | 42.8 | 2012-19 | 3 |
| Behcet Disease | Ireland | Adeeb 2018 | 2017 | pop | ≥18 | classification criteria | 6.2 |  |  |
|  | Norway | Lorentzen 2025 | 2021 | adm | any | ICD + review | 7.14 |  | 0.29 (0.18-0.41) |
|  | Poland | Kanecki 2017 | 2014 | adm | any | ICD hospitalized | 0.34 |  | 0.05 (0.03-0.06) |
|  | Spain | Suarez 2023 | 2018 | adm | any | diagnostic criteria | 10.1 |  | 0.49 |
|  | Sweden | Gisslander 2022 | 2019 | adm | ≥18 | ICD + diagnostic criteria | 9.1 (7.3-10.9 |  | 0.3 (0.2-0.3) |
|  | Switzerland | Villiger 2019 | 2015 | adm | any | diagnostic criteria | 4.03 |  |  |
|  | UK | Thomas 2020 | 2017 | adm | ≥18 | Read codes | 14.6 (13.4-15.9) |  | 0.61 (0.37-0.95) |
| Takayasu Arteriitis | Italy | Muratore 2021 | 2016 | pop | any | classification criteria | 0.9 |  |  |
|  | Poland | Kanecki 2018 | 2015 | adm | any | hospital based | 0.46 |  | 0.09 |
|  | Switzerland | Gloor 2021 | 2019 | adm | ≥18 | hospital, classification criteria | 1.5 |  | 0.03 |
|  | Turkey | Saritas 2016 | 2014 | adm | >16 | hospital based | 3.3 (1.9-4.8) |  |  |
| pop population-based study, adm administrative data, GC glucocorticoids, AID autoimmune disease, pSjD primary Sjoegren’s disease | | | | | | | | | |
